# How can engagement with underserved communities be enhanced? A co-inquiry informed model of stop smoking outreach

**DOI:** 10.1177/17579139251322314

**Published:** 2025-03-31

**Authors:** T Mills, L Dawkins, R Dean, EG Lewis, CL Jenkins, J Wills, S Sykes

**Affiliations:** PHIRST South Bank, London South Bank University, 103 Borough Road, London SE1 0AA, UK; PHIRST South Bank, London South Bank University, London, UK; Hertfordshire County Council, Stevenage, Hertfordshire, UK; PHIRST South Bank, London South Bank University, London, UK; PHIRST South Bank, London South Bank University, London, UK; PHIRST South Bank, London South Bank University, London, UK; PHIRST South Bank, London South Bank University, London, UK

**Keywords:** smoking cessation, community outreach, health inequalities, co-inquiry, complex intervention

## Abstract

**Aims:**

This co-inquiry project aimed to develop a qualitatively informed model of professionally led stop smoking outreach. It involved 13 staff from a Stop Smoking Service (SSS) which operates across three Local Authorities in England (Central Bedfordshire, Bedford Borough and Milton Keynes). Staff’s outreach sought to engage people from the most deprived areas who smoked but were not engaging with the service.

**Methods:**

The co-inquiry comprised six reflection sessions and ethnographic research which aimed to explicate and examine staff’s assumptions about how outreach works, conducted over 12 months. Data included 32 diary entries, eight observations of staff’s outreach events, 10 interviews with staff and eight interviews with members of the communities being targeted. Data were reflected on to develop a ‘real-world’ logic model and summarised using thematic analysis.

**Results:**

Professionally led outreach can raise awareness of service offers, remove access barriers and generate referrals. A non-judgemental, person-centred approach is vital through which staff carefully initiate conversations with community members about smoking, and tailor information to community members’ needs and preferences. Such an approach, in combination with an e-cigarette support option, can generate interest in SSS and challenge negative perceptions. However, outreach is time-consuming for busy frontline staff, unpredictable and best implemented via effective community partnerships.

**Conclusions:**

Our findings suggest that stop smoking advisors’ outreach can contribute substantially to national ambition to create a ‘smoke free generation’ provided that sufficient investment is provided. Professionally led outreach, delivered in partnership with community organisations, can generate referrals among people who are disconnected from health services. Such non-traditional referral routes are likely to become more significant as smoking prevalence further declines in the general population.

## Background

Smoking remains the leading cause of avoidable death and disease globally^1^ and a key driver of health inequality. Up to half the difference in death rates among men by socioeconomic status is attributable to smoking.^2–4^ In England and Wales, the proportion of people who smoke is up to four times higher in the most, compared to the least, deprived areas.^5,6^ Stop Smoking Services (SSS) in England, which provide freely available stop smoking support, have had considerable success in supporting people from disadvantaged groups and those with complex health needs.^5–7^ While rates of quit attempts are similar across socioeconomic groups, people of lower socioeconomic status are less likely to enact a successful quit attempt.^8,9^ Barriers to access include challenging life circumstances and features of SSS that make it harder for disadvantaged people to access.^10–13^ Despite these barriers, SSS’ greater reach among low socioeconomic groups has resulted in higher impact there compared to high socioeconomic groups, which suggests they have had a substantial impact on equity.^7^

Changing social norms and prevalence patterns, technological innovation (e.g. the arrival of e-cigarettes) and national policy developments are changing the landscape in which SSS operate. Recent funding increases, linked to the UK government’s ‘Stopping the Start’ policy strategy,^14^ follow a sustained period of budgetary cuts and local service reorganisations due to a combination of government austerity and declining footfall.^15^ NICE’s strategy for intensified, targeted focusing^16^ presents challenges for SSS because of the additional support needs of those groups who are most at risk of tobacco-related harm.^10^ With smoking prevalence declining in the general population, moreover, SSS may have to be more proactive to generate referrals from non-traditional routes. There is a need to elaborate on the role of SSS community outreach in this changing landscape.

International research on stop smoking community outreach is relatively sparse^17^ yet attests to its potential to engage otherwise hard-to-reach, or ‘underserved communities’, including ethnic groups known to encounter smoking-related health inequality,^18^ people with complex health needs^19^ and people from lower socioeconomic positions.^20^ In the UK, trial research of proactive smoking cessation supports the idea of outreach in a range of settings, including A&E^21^ and homeless centres.^22^ Client-led services, in which ‘trained lay advisors’ support community members to quit,^23^ as well as mobile drop-in services,^24^ have also demonstrated potential. And yet, there is a lack of research on community outreach by professional stop smoking advisors who are employed by SSS. Furthermore, while evidence suggests that e-cigarettes can work as an aid to quitting,^25^ the role of e-cigarettes in community outreach is only recently receiving research attention,^21,22^ requiring further elaboration.

### An evaluation of stop smoking community outreach

An SSS based across Bedfordshire Borough, Central Bedfordshire and Milton Keynes – referred to as ‘BMK’ – secured NIHR-funded evaluation assistance from PHIRST (Public Health Intervention Responsive Teams) South Bank. An evaluation design consisting of multiple work packages^26^ was coproduced by PHIRST South Bank researchers, BMK senior staff and a Patient Public Involvement and Engagement (PPIE) panel in three workshops: the PPIE panel provided a service-user perspective throughout the evaluation, providing guidance on the research focus, the design of data collection tools, recruitment (as panel members had links to the local area) and the presentation of findings.

This article presents the findings of a work package that aimed to understand and refine BMK’s efforts to raise awareness of the service through community outreach. Staff’s community outreach took place over 12 months immediately after COVID-19 restrictions eased. BMK had specialist support streams for people who smoke and are pregnant, under 18s and those with a mental health condition, learning disability or long-term physical condition. BMK staff wanted to explore if community outreach could enhance engagement with lower socioeconomic communities, in particular people from these communities who smoke but were not engaging with the SSS.

## Methods

A co-inquiry approach was adopted to formatively evaluate staff’s community outreach. Co-inquiry is a formative and participatory research method that can be used to generate learning and service improvement through facilitated reflections on professional practice.^27^ Here, the co-inquiry sought to understand and refine staff’s outreach efforts. A ‘real-world’ logic model^28^ was developed to support wider learning and scale-up activity.

Six co-inquiry sessions with 13 BMK staff were conducted over 12 months, running concurrently with staff’s outreach. A logic model was developed by T.M., who facilitated the co-inquiry, and refined through the sessions to elucidate staff’s evolving assumptions about what works in community outreach: for example, assumptions regarding staff’s strategies for initiating conversations with the public about smoking, or choice of outreach sites. T.M. undertook rapid ethnographic research to consider whether staff’s outreach worked as they anticipated, including observations of outreach events and interviews with community members. The latter were vital because, while the study had a PPIE panel who provided guidance throughout (see earlier), panel members were ex-service-users. We therefore sought community perspectives from those who were currently smoking and not engaging with BMK SSS (see Supplementary File 1 for the topic guide) to complement the PPIE panel. Staff members also kept diaries to record observations related to their outreach activities.

An initial data analysis phase occurred in real-time during the 12-month co-inquiry period. Written summaries of the data, including extracts of the observational data and quotations from interviews with community members, were presented to the PPIE panel and staff to make sense of the data dialogically^27^ and draw out implications for the logic model. This initial phase of data collection was iterative, providing a feedback loop that enabled informed discussion among staff and PPIE panel about what was happening in the field, as well as how data collection may be refined. Staff’s choice of outreach sites and the focus of the ethnographic research was informed through consensus discussion.

Finally, staff interviews were undertaken at the endpoint of the co-inquiry to explore individual views and experiences. All interviews were conducted online, recorded and transcribed. The full data set included the following:

- Thirty-two diary entries completed by staff over the 12 months.
- Observational field notes of eight outreach events observed by the research team.
- Ten semi-structured interviews with staff members.
- Eight semi-structured interviews with community members. Community members were recruited via outreach events or snowballing within the communities that staff sought to engage (see Table 1 for interviewee demographics).

**Table 1:** Community member demographics

Unique identifier	Sex	Age	Ethnicity
CM1	M	35	Black Caribbean
CM2	M	55	White British
CM3	M	37	White British
CM4	F	29	White British
CM5	F	38	White British
CM6	F	40	Asian British
CM7	M	62	White Irish
CM8	M	57	White British

Once the co-inquiry concluded, the research team undertook thematic analysis^29^ and real-world modelling^28^ to generate an in-context account of staff’s outreach. Interview data (from staff and community members) were organised and coded using NVivo 12. A coding framework was developed by T.M. and C.J. and applied to all interview data, with this supporting the identification of an initial set of themes inductively. These themes were then written out and refined via testing against the written summaries and observational extracts from the first data analysis phase (see earlier). Then, the themes and model were presented to BMK staff and the PPIE panel, with revisions made based on comments received. The final themes, presented below, were further edited and refined during the writing and review process.

## Findings

Data were organised into three themes which are presented first, followed by the real-world logic model. Illustrative quotes from the interview data are presented here; illustrative field notes can be found in Supplementary File 2:

Theme 1: Outreach-generated referrals: impactful yet unpredictable.
Theme 2: A person-centred offer with an e-cigarette option and community ties.
Theme 3: Navigating barriers and challenges during outreach delivery.

Theme 1 considers the main outcome of staff’s community outreach: referrals among underserved communities. Theme 2 identifies various contributory factors for outreach outcomes, namely staff’s ‘person-centred’ approach, an e-cigarette support option and community ties. Theme 3 then explores barriers and challenges that staff encountered during their outreach.

### Theme 1: Outreach-generated referrals: impactful yet unpredictable

Staff’s community outreach included 86 events over a 12-month period, with these generating referrals to the service that would not have occurred without the outreach. Service-users were assigned an appointment date for stop smoking support (in line with BMK SSS’ standard offer), or sometimes received a first appointment at the location of the outreach. Staff’s reflective diary entries, which described 32 of these events, reported 30 new referrals, suggesting an average of about one referral per event. As these referrals were generated during staff’s outreach in high deprivation areas, staff observed that they were likely to have implications for health inequalities related to smoking. Staff reported that some people they referred were disconnected from health services.

The outreach events were greatly appreciated by those who received a referral, with some highlighting how it had removed access barriers. For example, one community member who received an initial appointment at an event had difficulty moving due to a back injury and was unable to access stop smoking support via their GP:

*I couldn’t travel there to get the help . . . When you’re lucky enough to get in to see a GP it’s only one problem at a time, you can’t go in there with my back problem and then at the end of it, before I walk out, say ‘Oh, about smoking’ . . . It is all over the surgery, but once I’ve seen a doctor I just want to get out and get home so I can lay down and ease my back pain. (CM2)*

Outreach referrals were, however, highly variable, making it difficult to align staff presence with community demand. When attending alone, staff were sometimes unable to effectively deal with the demand at events while many other events did not result in any referrals. An indicator of this variation is the spread of referrals across staff’s reflective diary entries, with 16 of the 32 entries reporting zero referrals.

Meaningful contact with the public could occur without any referrals generated, prompting some staff to suggest that referrals should not be the sole focus. ‘Very Brief Advice’ was delivered in the form of conversations about the service offer as well as the health risks of smoking and e-cigarettes. Summer outreach events were attended by children, with various items (including a jar of tar and plastic smokers’ lungs) displayed, with possible preventive impacts:

*I got them to pick up the tar jar [and] discuss the lungs . . . We could focus on referrals which obviously is our main bread and butter, but also . . . [with] even that little bit of information, they might think twice if someone offers it to them. They might think: . . . ‘I remember the tar and the lungs, so I’m not going to bother’. (S3)*

In the absence of any public contact, outreach could still be considered useful by staff even though no referrals were generated. With outreach events often organised in collaboration with other organisations (e.g. local councils, Housing Associations, community organisations and health providers), staff often discussed with other staff groups strategies for facilitating connections with underserved communities. Staff also talked about the training and support they could provide if other staff wanted a direct role in delivering ‘Very Brief Advice’ or generating referrals, although this aspect of the outreach was aspirational, having paused during COVID-19.

### Theme 2: A person-centred offer with an e-cigarette option and community ties

Community members and most staff emphasised the importance of a person-centred, non-judgemental service offer. Staff had different strategies for initiating conversations about smoking which did not feature in national ‘Very Brief Advice’ training but had been developed through trial and error. One staff member explored neutral topics such as the weather or dogs, which tended to prompt community members to ask why they were there. The research team assessed this approach by interviewing a community member (CM2) who described it as ‘lovely’, with the staff member coming across as genuine and caring. However, the research team noted that one staff member’s jokey approach, which proceeded as if community members were being naughty if they smoked, clashed with the emphasis placed in co-inquiry sessions on the importance of a person-centred approach.

Staff offered advice and information that was tailored to community members’ circumstances, needs and preferences. Community members could opt for a carbon monoxide (CO)-read which provided immediate, personalised information on CO lung concentration, which could be a motivation to cut down or quit. A staff member describes their approach of encouraging community members to make an informed decision, with support immediately available should they decide to take it:

*I don’t push them into doing it . . . I will plant a seed . . . I did a CO read today . . . [with someone] who was doing the thin roll ups and . . . [a] vape. She said, ‘I think I’ll just stick with my vape’ and I said, ‘That’s fine, but let’s just do a CO read out of curiosity, and I’ll give you my card. You can always come back to me later’ . . . I did the CO read with her and it went red [indicating high CO concentration], and she said, ‘Right, where do I sign up? I want to quit’. (S8)*

The SSS’ new e-cigarette support offer, whereby service-users who are receiving support can choose to use an e-cigarette alongside standard Nicotine Replacement Therapies, generated interest and surprise among those community members who smoked but were not engaging. Among this group, the e-cigarette offer could serve as a conversation starter:

*Because the vapes were there . . . it made people more interested: ‘Why the hell are you doing that when there’s so much bad news around them?’ And then when you explained a bit more about them it opened it up a bit more. (S8)*

Staff dispelled misinformation about e-cigarettes – for example, that they cause ‘popcorn lung’ (CM3) – while making clear their view that e-cigarettes are a device to support quit attempts, not recreational use. Both staff and community members recognised the long-term uncertainties regarding the health implications of e-cigarettes. One community member agreed that it is legitimate for SSS to share those uncertainties with community members:

*By all means try and give out vapes to people . . . We won’t know [the full health implications] . . . for five or ten years . . . [but] it is worth it. It’s getting that balance right . . . There is no right or wrong and that’s why we’re having a debate now: it’s not black and white. (CM8)*

A contributory factor to successful outreach was ties with underserved communities that were being targeted, though these were challenging to establish by the SSS alone. One of the best performing outreach events – in terms of referrals generated – was organised by a Housing Association that had established connections with their tenants; one tenant was soon to take on a semi-formal role as a ‘community angel’ to raise awareness of events and encourage community members to attend. Various gifts, or ‘motivational pulls’, were also offered to interest community members and motivate them to talk with stop smoking staff. These included coffee and boxes of fruit and vegetables that were welcomed by community members, including those who were initially hostile to the idea of outreach, because ‘everyone likes something for free’ [CM5]. Community members warned staff, however, to be non-judgemental and not to impose themselves. Some community venues, such as local pubs, were considered out-of-bounds. In the following quote a community member advises that staff be present but do not ‘push out’:

*There needs to be a presence . . . they [staff] need to be there to be seen and be present . . . But don’t push out: wait to be approached . . . as soon as you push out, you’re preaching, and smokers are just going to close-up. (CM4)*

### Theme 3: Navigating barriers and challenges during outreach delivery

Staff agreed that the SSS should be more proactive in facilitating referrals and wanted to do outreach in high deprivation areas. However, many barriers were encountered, as staff sometimes struggled to reach community members due to poor event visibility. Some partner organisations lacked the organisational skills or access to popular locations to ensure events reached substantial numbers of people. Two staff members were frustrated at a school event, as the organiser requested their attendance at 12 noon, even after they had queried this start time. Staff then spent over 3 h waiting for parents to arrive. Such poor organisation could lead staff to question their involvement:

*Maybe it is just the events I’m attending, [but] we don’t seem to get [the] footfall and, in the end, I just end up helping the organisers . . . trying to get people off the street or knocking on doors or things like that, which isn’t really part of my job . . . You feel like, ‘Oh God, I’ve taken time out of my afternoon to come and sit at an event and it’s not worked’. (S9)*

The cost-of-living crisis presented opportunities and challenges, as people had more of an incentive to quit but economic stresses meant that some were less likely to, in a worrying dynamic recognised by staff and community members. Further challenges related to consumerism and populist beliefs, shared by some community members, that public health professionals constitute the ‘fun police’ [CM7]. One CM spoke about the camaraderie among smokers, as they are ‘being pushed into a corner’ [CM2]. Indeed, while welcome from a public health perspective, national policy restrictions on smoking were ambivalently viewed among community members. Some smokers viewed the restrictions as helping them control their habit and recognised the importance of denormalising smoking; others felt that the restrictions had gone ‘too far’ [CM2] and represented an ‘attack on freedom’ [CM3]. Community members also commonly knew where to purchase cheap cigarettes from the black market or from abroad. While such sociocultural dynamics present barriers, staff highlighted outreach’s vital contribution to public education, without which hostility may consolidate:

*From a communication and engagement point of view . . . if you do work on illicit tobacco and you go into the community and you take their cheap tobacco without any . . . [explanation] you are just going to create another, ‘Oh great, public health is coming in taking away my affordable tobacco, so what am I going to do now?’. So, it’s about helping people to understand the harms that come from this and the damage it does. (S10)*

### A complex intervention model of stop smoking outreach

**Figure 1:**
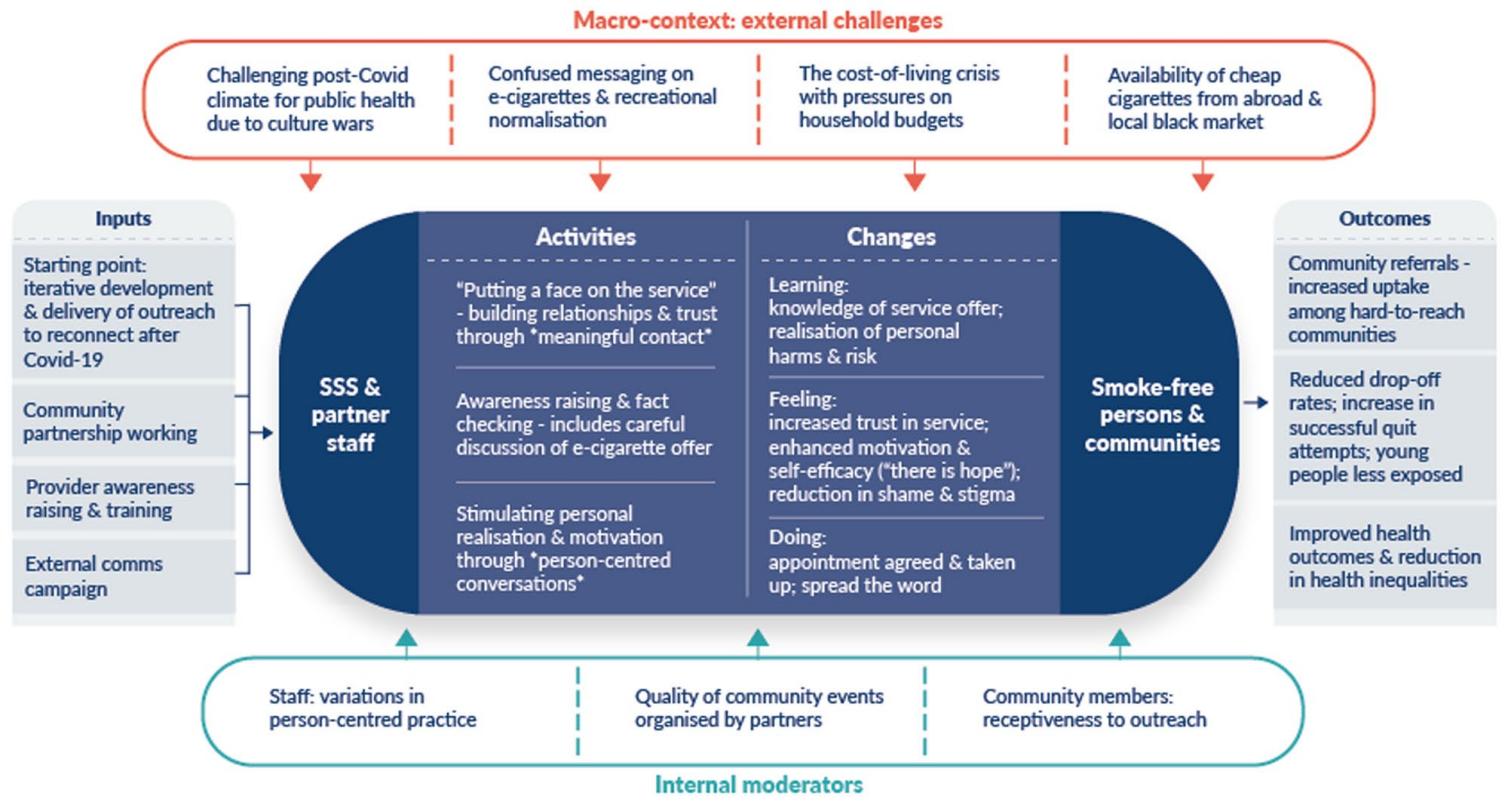
A complex intervention model of stop smoking outreach

Figure 1 presents a cross-theme summary model of the community outreach over the 12 months. The primary aim of staffs’ outreach was to generate referrals from underserved communities, as discussed in Theme 1; other outcomes included reduced drop off rates due to the service’s newfound immediacy, improved health outcomes among the individuals receiving a referral and possible reductions to smoking-related health inequality. These outcomes were achieved through various activities explored in Theme 2, which highlighted the importance of a positive, person-centred approach. Varied forms of individual-level change, categorised in terms of Learning, Feeling and Doing, were confirmed through the research. External challenges that hindered staff’s localised efforts to deliver outreach, explored in Theme 3, are listed at the top of the model. Factors at the local level that appeared to moderate implementation and outcomes internally, that is, linked to the people and organisations directly involved in the delivery and receipt of the outreach, are listed at the bottom. The main factor here was variability in the quality of outreach events organised by community partners; recognition of this led staff to be more strategic with regards to the events they attended and to collaborate with community partners to share best practices.

## Discussion

This article provides insight into the activities and impact of professionally led community outreach in a rapidly changing smoking landscape in England. We find that professionally led outreach that is delivered through effective community partnerships can raise awareness of SSS, remove access barriers and generate referrals. We further find that an e-cigarette offer generates interest in SSS among underserved communities, here substantiating staff’s desire to present a positive offer that countered negative perceptions of SSS as the ‘fun police’. This is significant because misconceptions about SSS, as well as access challenges, are known barriers to engagement.^30^ This article complements studies of proactive smoking cessation support which suggest that underserved communities respond to flexible service offers that ease the process of taking up support and include an e-cigarette offer.^21,22^ However, we also find that outreach is time-consuming for busy frontline staff and that the number of referrals generated is unpredictable. Outreach is best implemented via effective community partnerships to ensure that staff’s time is not wasted.

The UK government’s ‘Stopping the Start’ policy strategy announced £70 million per year increased funding to SSS (more than doubling previous expenditures), with a view to supporting 360,000 people to set a quit date annually. While this strategy recognises that support costs for the ‘most entrenched smokers’ are likely to rise,^14^ there is no consideration of access challenges, or the role of community outreach in addressing these. With smoking prevalence declining among the general population,^31^ non-traditional routes into SSS may become increasingly important as smoking concentrates among those with least contact with health services.^32^ We have suggested that professional stop smoking advisors can do community outreach alongside their core support work if appropriate investment is provided. Other evidence-based options include e-cigarette provision in A&E departments^21^ and homeless shelters,^22^ as well as client-led^23^ and mobile drop-in services.^24^ We recommend that some of the £70 million be utilised in forms of community outreach that are consistent with local need and resources.

Our findings resonate with broader arguments for brief advice delivery in line with the ‘Making Every Contact Count’ strategy,^33,34^ although the vital importance of measures to support people to act on health related information are highlighted here.^24,35^ Staff’s community outreach proactively facilitated referrals, with some community members receiving a first appointment on-site to enhance the accessibility of BMK SSS. The variability in staff’s person-centred practice that we identified, however, suggests the need for further interventional research to develop guidance and training in this area.

Our findings have implications for open questions about community organisations’ involvement in stop smoking support, as well as their place in the wider public health workforce.^36^ Research suggests that community organisations can facilitate engagement with larger and more diverse audiences than health professionals^33,37,38^ but may also face challenges due in part to knowledge and skills gaps.^39^ We find synergy in partnerships between professionally led SSS and community organisations, with the latter having a key role in organising public-facing events for professional staff to attend. It may be possible for community staff to take on a more direct role in brief advice delivery and referrals with the support of SSS, but this was only aspirational here.

## Strengths and Limitations

The strengths include the co-inquiry element which enabled the research team to develop close relationships with staff and to collaboratively assess their outreach over time. Diverse and detailed qualitative data facilitated deep insight and enabled triangulation, while the involvement of the PPIE panel brought different perspectives to the data that enhanced reflexivity through the research process. The limitations include this being a single, qualitative case study that involved one SSS (BMK) and occurred as COVID-19 restrictions were lifted. This is significant because other SSS teams may not have the resources or motivation to engage in community outreach, here prioritised due to senior managers’ perceiving that the service needed to reconnect with the public, and frontline SSS staff buying into this aim. Further limitations relate to the reach of the research and the size of our sample of community members. While SSS staff’s efforts successfully generated referrals among those disconnected from health services, and the research team were able to recruit a sample of eight members who lived in deprived areas, barriers were encountered to further engagement. More time in the field may have found more critical views among people who smoke but do not want contact with SSS while additional access barriers require further exploration. We were unable to explore language barriers in the current study.

## Conclusion

Stop smoking advisors have much to contribute to efforts to combat smoking harms beyond their core advisory role. Working in partnership with community organisations, they can generate referrals among people who are disconnected from health services. The complexity of outreach conversations, which require considerable sensitivity, indicate the importance of professional involvement in outreach, although further interventional research and training is required to standardise best practice. The time-consuming nature of outreach presents a major barrier, however, as frontline staff may struggle to combine outreach with their core advisory work without sufficient investment.

## Supplemental Material

sj-docx-1-rsh-10.1177_17579139251322314 – Supplemental material for How can engagement with underserved communities be enhanced? A co-inquiry informed model of stop smoking outreach Supplemental material, sj-docx-1-rsh-10.1177_17579139251322314 for How can engagement with underserved communities be enhanced? A co-inquiry informed model of stop smoking outreach by T Mills, L Dawkins, R Dean, EG Lewis, CL Jenkins, J Wills and S Sykes in Perspectives in Public Health

sj-docx-2-rsh-10.1177_17579139251322314 – Supplemental material for How can engagement with underserved communities be enhanced? A co-inquiry informed model of stop smoking outreach Supplemental material, sj-docx-2-rsh-10.1177_17579139251322314 for How can engagement with underserved communities be enhanced? A co-inquiry informed model of stop smoking outreach by T Mills, L Dawkins, R Dean, EG Lewis, CL Jenkins, J Wills and S Sykes in Perspectives in Public Health
