# Supplementary material for: How can engagement with underserved communities be enhanced? A co-inquiry informed model of stop smoking outreach
Source: Perspect Public Health. 2025 Mar 31;145(2):97–104. doi: 10.1177/17579139251322314 (PMC12069823; doi:10.1177/17579139251322314)
Supplement: sj-docx-1-rsh-10.1177_17579139251322314 – Supplemental material for How can engagement with underserved communities be enhanced? A co-inquiry informed model of stop smoking outreach [file sj-docx-1-rsh-10.1177_17579139251322314.docx]

**Pre-chat – introduce researcher and purpose of the interview**

-We are conducting research into outreach initiatives being delivered by BMK Stop Smoking Service (SSS). You have been identified as a person who lives in a community that BMK SSS’ outreach is aiming to engage. And you smoke but you are not currently engaging with the service.

-We would like to ask you a few questions about your smoking, awareness of the outreach initiatives and your thoughts on whether you think they have been appropriately designed and delivered. We will describe these to you if you have no awareness of them.

-We have already taken your informed consent. As a reminder, the interview is being audio recorded for research purposes only; interviewees are free to withdraw at any time; if there are questions which you don’t like, or an area you don’t want to discuss, then please say so and we will move on; everything said is confidential and interview data will be anonymised

-Any questions before we start? Confirm if happy to go ahead and start recording

**Introduction and questions about smoking**

- Can you tell be a bit of about yourself? (Depending on the target group of the outreach initiative, prompt for: work role, postcode or ethnic group)
- How would you describe your smoking? (Prompt for: heavy or light, occasional or regular, individual or social)
- When/where do you smoke? (Prompt for: home, workplace, journey to and from work, at a friend’s)
- Why do you smoke? (Prompt for: when started or now)
- Is there anything that triggers you to smoke? (Prompt for: boredom, stress, socialising, drinking, family influence)
- Are you motivated to quit? Prompt for: easy to quit or difficult, attempted before or never attempted, reason for abeyance)

**Awareness of and access to Stop Smoking Service**

- If you wanted support to quit smoking, where would you go for help?
- Are you aware that [enter council name] offers support to help you quit?
- What barriers are there to you accessing this service? Are these the same for peers, or family members who smoke?
- The SSS service has recently moved to telephone delivery: does that make it more or less likely that you/your peers/your family members will seek their support? Why?

**Perception and impact of outreach initiative** (to be asked if interviewee attended an outreach event or has received an outreach intervention component – e.g., a leaflet)

- How did you find the [refer to outreach initiative or intervention]?
- Did you learn anything from it? What? [Prompt for: smoking harms, awareness of support available, how to access the support]
- Have you shared your learning among peers or family members?
- How, if at all has the outreach initiative impacted on your motivation to quit?
- Have you sought help since attending/receiving the event/intervention? Will you seek help in the future?
- How might the outreach event/intervention be enhanced? (Prompt for: reach out to your peers or family members who smoke)

**Possible impact of outreach initiative** (to be asked if interviewee has not attended an outreach event or received an outreach intervention component)

- How might [enter council name] SSS reach out to you to inform you about the support available to you? (Prompt for: how to reach out to peers, colleagues, friends or family members who smoke)
- [Enter council name] SSS is currently [describe outreach initiative or present intervention component]: what do you think about that approach/this intervention component?
- Do you think you would learn something from this event/intervention if you attended/received it? What? [Prompt for: smoking harms, awareness of support available, how to access the support]
- How might it impact on your motivation to quit?
- How might the event/intervention be improved?
- What barriers are there to your peers/family members attending this event/receiving this intervention?
