# Supplementary material for: How can engagement with underserved communities be enhanced? A co-inquiry informed model of stop smoking outreach
Source: Perspect Public Health. 2025 Mar 31;145(2):97–104. doi: 10.1177/17579139251322314 (PMC12069823; doi:10.1177/17579139251322314)
Supplement: sj-docx-2-rsh-10.1177_17579139251322314 – Supplemental material for How can engagement with underserved communities be enhanced? A co-inquiry informed model of stop smoking outreach [file sj-docx-2-rsh-10.1177_17579139251322314.docx]

**Supplementary File 2**

This Supplementary File presents illustrative fields notes underpinning the three themes. The field notes were written by members of the research team after they attended outreach events (see Table 1).

**Table 1: Outreach events**

| **Observational event** | |
| --- | --- |
| 1 | Housing Association |
| 2 | Community event (open air) |
| 3 | Supermarket |
| 4 | Housing Association |
| 5 | School |
| 6 | Community event (indoor) |
| 7 | GP practice |
| 8 | Housing Association |

**Theme 1: Outreach-generated referrals: impactful yet unpredictable**

The following extract describes a successful outreach event. One person received their first support session at a Housing Association building (“the Hub”) on the estate where they lived. A second person came to speak to the stop smoking advisor, but they were unavailable because the advisor was delivering the support session:

*Probably after 1 hour [of being there], a man came who wanted to quit smoking (Client A). They reported “going through so much” at the moment, with health issues and other life problems … and walked in a lot of pain. They were planning to quit but they didn’t realise that support was available. They only realised this from seeing the event and inquiring about what was on offer.*

*A stop smoking advisor went with the man to “the Hub” to book them in. The advisor explained that it is preferable to sign people up and give them an appointment on the spot because, if they are provided with leaflets, people might not sign up themselves. The advisor took a long time doing this and … later explained that they had booked them in and then done the first support session with them, as they had the time and opportunity. They had done the first session and carried out a carbon monoxide test.*

*The advisor wasn’t aware that, as they were doing the appointment, another man had driven up and spotted the poster (Client B). The driver screeched to a halt and asked questions from the window. They pointed to the poster saying, “that is what I need”. Their father had had a heart attack and they wanted to give up smoking to encourage their father to give up smoking. After a brief discussion from the window of the car, they parked up and got out to chat further. They didn’t have time to wait [for the advisor] because they had to pick up their mother to take her to the hospital to visit their father. We gave them a leaflet.*

*I said to a Housing Association staff member “there’s some demand for the service”, and they agreed. It may also highlight a resourcing issue. The advisor had been keen to do the appointment with Client A but that meant the advisor wasn’t available to book Client B onto the system. They were given a leaflet, but this leaves it up to the client to sign up, which is known to sometimes not work. Having two advisors on the stand would solve this, but there wasn’t enough staff to cover [Field notes: event 1].*

The following extract describes an event at a GP surgery that was unsuccessful at generating referrals. The stop smoking advisor was surprised because events at GP surgeries had generated referrals in the past:

*[Redacted name of stop smoking advisor] suggested I attend the [Redacted name of GP practice] event because, on paper, the outreach event looked promising. There was a school nearby, a pharmacy next to the GP surgery and a pub: the pharmacy, GP surgery and pub all shared the same car park. On paper, therefore, the event had potential to reach people and I prioritised this over other suggested events. There was an employment event running on the same day that was suggested but I was emailed to say that this might not be useful because it was aimed at employers rather than the public …*

*The stall was in the entrance to the general practice and had poor visibility, both for people inside the practice or passers-by. There were no contacts with smokers apart from a member of staff. No one had a CO test. No one took a leaflet.* *[Redacted name of stop smoking advisor] explained that previously there were usually lots of contact, when general practices were busy and people waited in the waiting room. [Redacted name of stop smoking advisor] told a story when people used to wait outside smoking and sheepishly smile as they would hide a cigarette, knowing that [Redacted name of stop smoking advisor] would speak to them when they returned to the practice. [Redacted name of stop smoking advisor] then pointed around saying “look, there is no one” [Field notes: event 7]*

A previously successful Housing Association event did not generate referrals on the researcher’s second visit but did result in many public contacts:

*This was my second [redacted name of road] visit … It was good to see [redacted name of stop smoking advisor] again and [redacted name of a local council coordinator], who collaborated with the Housing Association to put on the event. The event was well-attended: probably 30 points of contact in total but no referrals were generated. A local smoker did stop by and chatted to [redacted name of a local council coordinator]. Unfortunately, [redacted name of stop smoking advisor] was busy with someone else so they weren’t able to chat directly to the smoker but [redacted name of a local council coordinator] passed them flyers. [redacted name of a local council coordinator] also explained that [redacted name of stop smoking advisor] would be back in a few weeks’ time so they could wait until then [Field notes: event 8]*

Other events, while failing to generate new referrals, could be useful for staff as they presented networking opportunities with other provider staff:

*While the primary purpose of the event was to reach people from deprived communities, it was mainly working as a networking and raising awareness opportunity among the various health teams in attendance. [redacted name of stop smoker advisor] spotted a substance use worker who had had some initial stop smoking training and chatted with them to remind them of the service, see how it was going for them, and to see whether they needed any resources. Other health workers came and chatted, taking details of the service. One health worker commented that they weren’t aware of it. They were impressed and would explore taking up the training offer. The fact that the event was in a single room helped this because everyone was pushed into close proximity and it was impossible to not start up conversations with people [Field notes: event 6].*

**Theme 2: A person-centred offer with an e-cigarette option and community ties**

The following extract describes a stop smoking advisor’s approach to outreach:

*[Redacted name of stop smoking advisor]’s approach was to initiate contact by talking about neutral topics, for example the weather or dogs. The conversation seemed to then naturally flow onto the reason for the outreach event and smoking. One person [redacted name of stop smoking advisor] talked to was not a smoker but they had a family member who smoked and said they would give them a leaflet. It was very natural …*

*At one point, a mother and two children came over to the smoking stall. She had previously quit smoking and explained that she wanted to quit vaping too. [Redacted name of stop smoking advisor] explained that they could help them in that. Her kids were interested in the display items and were interested in the various toxins that were displayed in the box. It was as if the mother was using the display to reinforce the message that she had been telling her kids. They appeared genuinely interested and one said they would never smoke [Field notes: event 4].*

The following extract describes a stop smoking advisor’s “paternalistic” approach to outreach:

*[Redacted name of stop smoking advisor] is assertive and quite paternalistic in their approach to outreach. They recalled a situation when, whilst queuing in a newsagents, a customer complained that a lighter they had been sold had broken, prompting them to say: “Well, you should give up smoking then, shouldn’t you?” This may have been a friendly encounter, but I wondered what that person would have taken away from the encounter: it seems to clash with the sense of an unassuming offer that the outreach work is seeking to convey ... [Redacted name of stop smoking advisor] has good rapport with people but it’s sometimes as if people are being told off for doing something naughty [Field notes: event 3]*

The following extract describes a “friendly conversation” about e-cigarettes between a stop smoking advisor and people queuing for a pharmacy to open. The stop smoking advisor clarified to the researcher that e-cigarettes are not promoted as a lifestyle choice:

*There was a queue for the pharmacy and [Redacted name of stop smoking advisor] engaged with them in friendly conversation. They mainly had questions about vaping and clarified things about the relative risk, problems with unregulated vaping products and potential for children to be interested …*

*We discussed vaping after which was interesting. I queried whether offering this might open up new opportunities to engage those who are difficult to engage. She made it very clear that the vaping option is only offered to help them quit the smoking, and that, in a 12-week programme, they will then have to quit vaping themselves, or buy vaping products, because they’re unlikely to quit both smoking and vaping within that period: vaping is not being offered as a lifestyle alternative that smokers will be supported to adopt [Field notes: event 7]*

Community members were surprised by the stop smoking service’s offer of support using an e-cigarette:

*The smoker could not believe that the service offered a vape. They were quite taken aback [Field notes: event 8]*

The following field diary extract describes the use of “motivational pulls”:

*The fruit n veg boxes were a giveaway to attract people. There was some discussion about whether it looked like a market stall and the boxes were for sale (this had been my initial interpretation, when walking around). They’d previously had a tea and coffee van and someone from the neighbourhood had remarked, when they were setting up, “there’s the coffee people”. Apparently, there had been a queue of people then, but the tea and coffee van couldn’t come this time. Over the 2 hours we were there, I counted 12 people come to take away the fruit and veg boxes. One person joked about how their child loves eating fruit and vegetables. Some people also came over to chat to the stop smoking advisor who weren’t interested in the box [Field notes: event 1]*

The following extract describes the good practices of one Housing Association:

*A woman who lived over the road came over to say “hello” – I did not catch her name. Someone explained that the woman had given up smoking some time ago and were now helping the Housing Association and other health teams to link into the community. While at the stall, the woman called over to a couple of people who smiled although they didn’t come over because they were busy. She conveyed that this was a friendly stall. A Housing Association staff member explained that the woman may be taking on a semi-official role in future as a “community angel” …*

*I chatted with* *[redacted name of Housing Association staff member] who was the lead for the Housing Association. They have public health expertise, having done a Master’s in Public Health …. [and] talked about this being new for the Housing Association, and there being variability among Housing Associations, with [redacted name of Housing Association] being somewhat advanced … I would later learn that this is a national policy development and Housing Association KPIs map those with local councils/public health departments [Field notes: event 8]*

**Theme 3: Navigating barriers and challenges during outreach delivery**

The following extract identifies challenges to outreach related to the visibility of the events:

*The building was not situated centrally in the neighbourhood but on a main “through” road. It was set a little bit back and the cars of the various health worker teams were parked up alongside it which obscured the view of the building. While the various teams had put posters up, for people driving past these were difficult to see. Most of the activity was inside the building which obscured things further. It was striking that, as I left, I saw a car drive past and the driver was smoking in the window. They may not have wanted to quit but, even if they did, they were unlikely to have known about the stop smoking event because it lacked visibility from the road [Field notes: event 4]*

The following extract describes a school event at which two stop smoking advisors waited for over 3 hours for the event to start:

*I chatted with [redacted name of stop smoker advisor] and [redacted name of stop smoker advisor] at length as we waited for the event to begin. We talked about the project generally and the event. They were quietly annoyed at the situation because they had queried the 12 noon start time, but the organiser had insisted that it would start then. But the event only got going when the parents arrived to pick up their children at 3:15pm, when it got busy and the advisors spent time chatting to people, answering questions and doing CO-reads. It definitely warranted two people as there were many people to talk to, and advisors appear to enjoy it more when they do it with colleagues. But [redacted name of stop smoker advisor] and [redacted name of stop smoker advisor] talked about the difficulty fitting the outreach into their busy work schedules and they were clearly frustrated with having to wait [Field notes: event 5].*

The following extract describes a poorly organised event as well as challenges linked to poor weather:

*There were no referrals from this event. Very few people approached and there was little interaction between staff although [redacted name of stop smoking advisor] did pop around and say “hello” to the health workers present: there was about five, including a weight management team and a sports team. The stalls were situated in an open park so there was good visibility but the only people that came by were people on a walk or dog walkers: it wasn’t a busy road with high footfall. Also, it rained: this didn’t help as there may have been more people through the day if it was sunnier, and a downfall at around 2pm meant closing the stall down altogether. Finally, there wasn’t much interaction between teams because they were far apart from each other.*

*I had an interesting conversation with a health worker who questioned the event design. I approached them and chatted to them about the event. We talked about the lack of people and they questioned the choice of location and the timing of the event. They pointed to the nearest houses (about 200 metres away) and said, “Look at those. They’re well-to-do houses with separate drives”. They also said that people are probably on their summer holidays so they may not be around. They suggested that it would be better in [redacted name of a local area], as this was a more deprived area, and there were grassy areas between rows of houses where the stalls could be set up [Field notes: event 2].*
